# 3′-(4-Chloro­benzo­yl)-1′-methyl-4′-[5-(2-thien­yl)-2-thien­yl]spiro­[acenaphthyl­ene-1,2′-pyrrolidin]-2(1*H*)-one

**DOI:** 10.1107/S1600536810053870

**Published:** 2011-01-08

**Authors:** S. Thenmozhi, E. Govindan, D. Gavaskar, R. Raghunathan, A. SubbiahPandi

**Affiliations:** a Department of Physics, Presidency College (Autonomous), Chennai 600 005, India; b Department of Organic Chemistry, University of Madras, Guindy Campus, Chennai 600 025, India

## Abstract

In the title compound, C_31_H_22_ClNO_2_S_2_, the five-membered pyrrolidine ring, which exhibits an envelope conformation, makes a dihedral angle of 87.4 (2)° with the acenaphthyl­ene ring system. The crystal structure is stabilized by π–π inter­actions [centroid–centroid distance = 3.869 (2) Å]. A C atom and the S atom of the thiophene ring are disordered over two positions with refined occupancies of 0.629 (7) and 0.372 (7).

## Related literature

For general background to the applications and biological activity of the title compound, see: Sarala *et al.* (2006 ▶). For puckering parameters, see: Cremer & Pople (1975 ▶) and for asymmetry parameters, see: Nardelli *et al.* (1983 ▶).
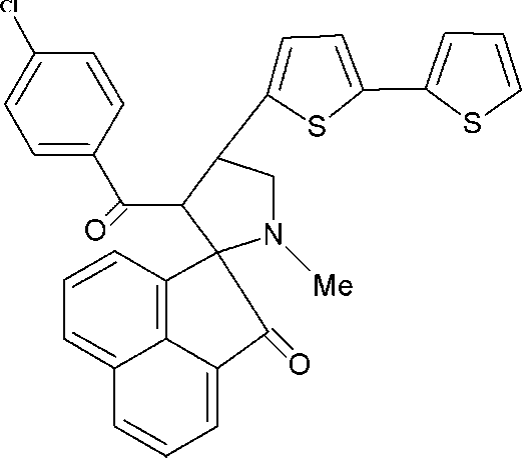


## Experimental

### 

#### Crystal data

C_31_H_22_ClNO_2_S_2_
*M* *_r_* = 540.07
Orthorhombic, 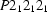

*a* = 12.6858 (13) Å
*b* = 13.6733 (13) Å
*c* = 15.2782 (17) Å
*V* = 2650.1 (5) Å^3^
*Z* = 4
Mo *K*α radiation
μ = 0.33 mm^−1^
*T* = 293 K
0.25 × 0.22 × 0.19 mm

#### Data collection

Bruker APEXII CCD area detector diffractometer
Absorption correction: multi-scan (*SADABS*; Sheldrick, 2006 ▶) *T*_min_ = 0.920, *T*_max_ = 0.939
13196 measured reflections
4384 independent reflections
3364 reflections with *I* > 2σ(*I*)
*R*_int_ = 0.033

#### Refinement

*R*[*F* ^2^ > 2σ(*F* ^2^)] = 0.045
*wR*(*F* ^2^) = 0.113
*S* = 1.04
4384 reflections
355 parameters
5 restraints
H-atom parameters constrained
Δρ_max_ = 0.31 e Å^−3^
Δρ_min_ = −0.28 e Å^−3^
Absolute structure: Flack (1983 ▶), 1898 Friedel pairs
Flack parameter: 0.00 (9)

### 

Data collection: *APEX2* (Bruker, 2004 ▶); cell refinement: *SAINT* (Bruker, 2004 ▶); data reduction: *SAINT*; program(s) used to solve structure: *SHELXS97* (Sheldrick, 2008 ▶); program(s) used to refine structure: *SHELXL97* (Sheldrick, 2008 ▶); molecular graphics: *ORTEP-3* (Farrugia, 1997 ▶); software used to prepare material for publication: *SHELXL97* and *PLATON* (Spek, 2009 ▶).

## Supplementary Material

Crystal structure: contains datablocks global, I. DOI: 10.1107/S1600536810053870/bt5403sup1.cif

Structure factors: contains datablocks I. DOI: 10.1107/S1600536810053870/bt5403Isup2.hkl

Additional supplementary materials:  crystallographic information; 3D view; checkCIF report

## Figures and Tables

**Fig. 1.**
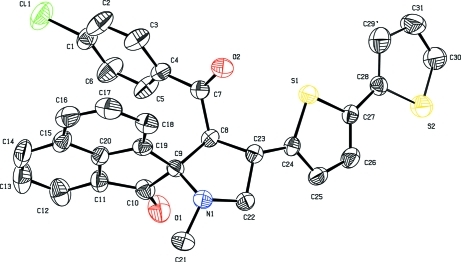
The structure of showing the atom-numbering scheme and intramolecular hydrogen bond. Displacement ellipsoids are drawn at the 30% probability level.For clarity H atoms are omitted.

## supplementary crystallographic information

### Comment

X-Ray analysis confirms the molecular structure and atom connectivity as illustrated in Fig. 1.The geometric parameters in the title compound agree with the reported values of a similar structure (Sarala *et al.*, 2006). The pyrrolidine ring makes dihedral angles of 42.3 (2), 89.8 (2) and 87.5 (1)° with the acenaphthylene ring system and the phenyl ring, and bithiophene rings respectively. The sum of the angles at N1 of the pyrrolidine ring (341.1°) is in accordance with *sp*^3^ hybridization. The pyrrolidine ring (N1/C9/C8/C23/C22) adopt an envelope conformation, with the puckering parameters q_2_ and φ (Cremer & Pople, 1975) and the smallest displacement asymmetric parameters, Δ, (Nardelli *et al.*, 1983) as follows: q_2_= 0.410 (3) Å, φ= 316.4 (5)°, Δ_s_(C9)= 4.2 (3)°.The thiophene ring (S2/C28/C29'/C31/C30) adopt an envelope conformation, with the puckering parameters q_2_ and φ (Cremer & Pople, 1975) and the smallest displacement asymmetric parameters, Δ, (Nardelli *et al.*, 1983) as follows: q_2_= 0.062 (7) Å, φ= 357 (12)°, Δ_s_(C29')= 1.1 (12)°.

The molecular structure of the title compound shows two intramolecular hydrogen bonds. The crystal packing is stabilized by π–π electron interactions. The π–π interactions between the rings *Cg*4 - *Cg*6 at *x*, *y*, *z* with the centroid-centroid distance equal to 3.869 (2) Å, is observed in the crystal structure [*Cg*4 and *Cg*6 are the centroids of the rings C9/C10/C11/C19/C20 and C1—C6].

### Experimental

A solution of the (4-chloro-phenyl-3-Bithiophenyl-prop-2-ene-1-one derived from Bithiophene (1- mmol), Acenapthoquinone (1 mmol), sarcosine (1 mmol) in toluene (30 ml) was refluxed for 8 hrs. The progress of the reacion was evidenced by the TLC analysis. The solvent was removed under reduced pressure and the crude product was subjected to column chromatogarphy using petroleum ether/ethyl acetate (4:1) as solvent. X-ray diffraction were obtained by slow evaporation of a solution of the title compound in hexene at room temperature.

### Refinement

The C and S atoms of the thiophene ring are disordered over two positions (C29/C29' and S2/S2') with refined occupancies of 0.629 (7) and 0.373 (7). The corresponding bond distances involving the disordered atoms were restrained to be equal, and also the same *U*^ij^ parameters were used for atoms C29 and C29'and S2 and S2'. All H atoms were fixed geometrically and allowed to ride on their parent C atoms, with C—H distances fixed in the range 0.93–0.97 Å with *U*_iso_(H) = 1.5*U*_eq_(C) for methyl H 1.2*U*_eq_(C) for other H atoms.

### Crystal data

**Table:**

C_31_H_22_ClNO_2_S_2_	*F*(000) = 1120
*M**_r_* = 540.07	*D*_x_ = 1.354 Mg m^−^^3^
Orthorhombic, *P*2_1_2_1_2_1_	Mo *K*α radiation, λ = 0.71073 Å
Hall symbol: P 2ac 2ab	Cell parameters from 4384 reflections
*a* = 12.6858 (13) Å	θ = 2.0–24.5°
*b* = 13.6733 (13) Å	µ = 0.33 mm^−^^1^
*c* = 15.2782 (17) Å	*T* = 293 K
*V* = 2650.1 (5) Å^3^	Block, colourless
*Z* = 4	0.25 × 0.22 × 0.19 mm

### Data collection

**Table:**

Bruker APEXII CCD area detector diffractometer	4384 independent reflections
Radiation source: fine-focus sealed tube	3364 reflections with *I* > 2σ(*I*)
graphite	*R*_int_ = 0.033
ω and φ scans	θ_max_ = 24.5°, θ_min_ = 2.0°
Absorption correction: multi-scan (*SADABS*; Sheldrick, 2006)	*h* = −14→14
*T*_min_ = 0.920, *T*_max_ = 0.939	*k* = −15→12
13196 measured reflections	*l* = −17→17

### Refinement

**Table:**

Refinement on *F*^2^	Secondary atom site location: difference Fourier map
Least-squares matrix: full	Hydrogen site location: inferred from neighbouring sites
*R*[*F*^2^ > 2σ(*F*^2^)] = 0.045	H-atom parameters constrained
*wR*(*F*^2^) = 0.113	*w* = 1/[σ^2^(*F*_o_^2^) + (0.0474*P*)^2^ + 0.7498*P*] where *P* = (*F*_o_^2^ + 2*F*_c_^2^)/3
*S* = 1.04	(Δ/σ)_max_ = 0.001
4384 reflections	Δρ_max_ = 0.31 e Å^−^^3^
355 parameters	Δρ_min_ = −0.28 e Å^−^^3^
5 restraints	Absolute structure: Flack (1983), 1898 Friedel pairs
Primary atom site location: structure-invariant direct methods	Flack parameter: 0.00 (9)

### Special details

**Table:**

Geometry. All e.s.d.'s (except the e.s.d. in the dihedral angle between two l.s. planes) are estimated using the full covariance matrix. The cell e.s.d.'s are taken into account individually in the estimation of e.s.d.'s in distances, angles and torsion angles; correlations between e.s.d.'s in cell parameters are only used when they are defined by crystal symmetry. An approximate (isotropic) treatment of cell e.s.d.'s is used for estimating e.s.d.'s involving l.s. planes.
Refinement. Refinement of *F*^2^ against ALL reflections. The weighted *R*-factor *wR* and goodness of fit *S* are based on *F*^2^, conventional *R*-factors *R* are based on *F*, with *F* set to zero for negative *F*^2^. The threshold expression of *F*^2^ > σ(*F*^2^) is used only for calculating *R*-factors(gt) *etc*. and is not relevant to the choice of reflections for refinement. *R*-factors based on *F*^2^ are statistically about twice as large as those based on *F*, and *R*- factors based on ALL data will be even larger.

### Fractional atomic coordinates and isotropic or equivalent isotropic displacement parameters (Å^2^)

**Table:**

	*x*	*y*	*z*	*U*_iso_*/*U*_eq_	Occ. (<1)
C1	1.3937 (4)	−0.2222 (3)	0.0631 (3)	0.0800 (13)	
C2	1.3483 (5)	−0.2581 (3)	0.1358 (4)	0.1047 (17)	
H2	1.3714	−0.3174	0.1586	0.126*	
C3	1.2680 (4)	−0.2083 (3)	0.1769 (3)	0.0861 (13)	
H3	1.2359	−0.2350	0.2261	0.103*	
C4	1.2353 (3)	−0.1191 (3)	0.1455 (2)	0.0566 (9)	
C5	1.2854 (4)	−0.0835 (3)	0.0728 (3)	0.0832 (13)	
H5	1.2662	−0.0223	0.0514	0.100*	
C6	1.3632 (4)	−0.1351 (3)	0.0302 (3)	0.0966 (16)	
H6	1.3942	−0.1104	−0.0204	0.116*	
C7	1.1509 (3)	−0.0646 (2)	0.1911 (2)	0.0533 (8)	
C8	1.1575 (3)	0.0467 (2)	0.1911 (2)	0.0472 (8)	
H8	1.1689	0.0684	0.1307	0.057*	
C9	1.2525 (3)	0.0844 (2)	0.2484 (2)	0.0496 (8)	
C10	1.3424 (3)	0.1227 (3)	0.1863 (2)	0.0624 (10)	
C11	1.4414 (3)	0.0747 (3)	0.2121 (2)	0.0654 (10)	
C12	1.5430 (4)	0.0814 (4)	0.1802 (3)	0.0920 (15)	
H12	1.5600	0.1236	0.1346	0.110*	
C13	1.6186 (4)	0.0224 (5)	0.2192 (4)	0.1115 (19)	
H13	1.6874	0.0257	0.1985	0.134*	
C14	1.5970 (4)	−0.0401 (4)	0.2861 (4)	0.1040 (18)	
H14	1.6512	−0.0773	0.3101	0.125*	
C15	1.4942 (4)	−0.0495 (3)	0.3196 (3)	0.0740 (12)	
C16	1.4591 (5)	−0.1096 (3)	0.3881 (3)	0.0922 (15)	
H16	1.5067	−0.1504	0.4166	0.111*	
C17	1.3559 (5)	−0.1086 (3)	0.4134 (3)	0.0899 (15)	
H17	1.3347	−0.1489	0.4592	0.108*	
C18	1.2801 (3)	−0.0481 (3)	0.3719 (2)	0.0705 (11)	
H18	1.2103	−0.0486	0.3905	0.085*	
C19	1.3106 (3)	0.0107 (2)	0.3048 (2)	0.0552 (9)	
C20	1.4180 (3)	0.0097 (3)	0.2798 (2)	0.0565 (8)	
C21	1.2724 (4)	0.2350 (3)	0.3369 (3)	0.0880 (13)	
H21A	1.2967	0.2752	0.2895	0.132*	
H21B	1.3318	0.2051	0.3653	0.132*	
H21C	1.2348	0.2746	0.3783	0.132*	
C22	1.1089 (3)	0.1941 (2)	0.2613 (2)	0.0567 (9)	
H22A	1.1251	0.2389	0.2139	0.068*	
H22B	1.0624	0.2262	0.3027	0.068*	
C23	1.0607 (3)	0.1006 (2)	0.2272 (2)	0.0496 (8)	
H23	1.0329	0.0634	0.2769	0.060*	
C24	0.9739 (3)	0.1152 (2)	0.1621 (2)	0.0508 (8)	
C25	0.9350 (3)	0.1998 (3)	0.1294 (2)	0.0663 (10)	
H25	0.9606	0.2610	0.1453	0.080*	
C26	0.8532 (3)	0.1871 (3)	0.0695 (3)	0.0721 (11)	
H26	0.8194	0.2393	0.0423	0.086*	
C27	0.8272 (3)	0.0943 (3)	0.0545 (2)	0.0522 (8)	
C28	0.7479 (3)	0.0563 (3)	−0.0029 (2)	0.0568 (9)	
C30	0.6091 (4)	0.0442 (3)	−0.1079 (3)	0.0979 (16)	
H30	0.5593	0.0565	−0.1511	0.117*	
C31	0.6363 (4)	−0.0460 (4)	−0.0872 (4)	0.111 (2)	
H31	0.6068	−0.1032	−0.1091	0.133*	
N1	1.2030 (2)	0.1596 (2)	0.30319 (19)	0.0615 (8)	
O1	1.3271 (2)	0.1816 (2)	0.1289 (2)	0.0946 (10)	
O2	1.0810 (2)	−0.10617 (19)	0.23110 (18)	0.0746 (7)	
S1	0.90714 (8)	0.01840 (7)	0.11587 (6)	0.0631 (3)	
S2	0.6657 (4)	0.1332 (3)	−0.0552 (3)	0.0847 (11)	0.629 (7)
C29'	0.7190 (17)	−0.0390 (8)	−0.0252 (15)	0.105 (8)	0.629 (7)
H29'	0.7515	−0.0937	−0.0011	0.126*	0.629 (7)
S2'	0.7253 (10)	−0.0658 (7)	−0.0140 (8)	0.097 (2)	0.372 (7)
C29	0.686 (2)	0.1034 (13)	−0.0665 (19)	0.128 (16)	0.372 (7)
H29	0.6957	0.1690	−0.0806	0.153*	0.372 (7)
Cl1	1.49143 (12)	−0.28892 (10)	0.00879 (10)	0.1206 (6)	

### Atomic displacement parameters (Å^2^)

**Table:**

	*U*^11^	*U*^22^	*U*^33^	*U*^12^	*U*^13^	*U*^23^
C1	0.084 (3)	0.079 (3)	0.077 (3)	0.026 (3)	−0.020 (3)	−0.028 (2)
C2	0.144 (5)	0.069 (3)	0.102 (4)	0.045 (3)	−0.011 (4)	0.006 (3)
C3	0.118 (4)	0.062 (3)	0.078 (3)	0.018 (3)	0.003 (3)	0.005 (2)
C4	0.072 (2)	0.048 (2)	0.050 (2)	0.0084 (18)	−0.0128 (18)	−0.0038 (16)
C5	0.117 (4)	0.064 (3)	0.068 (3)	0.032 (3)	0.016 (3)	0.003 (2)
C6	0.128 (4)	0.082 (3)	0.080 (3)	0.032 (3)	0.027 (3)	0.001 (3)
C7	0.060 (2)	0.054 (2)	0.0459 (19)	−0.0004 (18)	−0.0096 (17)	0.0039 (16)
C8	0.056 (2)	0.0465 (18)	0.0389 (16)	0.0059 (16)	0.0012 (15)	0.0043 (14)
C9	0.047 (2)	0.0477 (18)	0.0542 (19)	−0.0028 (16)	−0.0017 (15)	0.0025 (15)
C10	0.060 (2)	0.065 (2)	0.062 (2)	−0.006 (2)	0.0040 (19)	0.008 (2)
C11	0.050 (2)	0.080 (3)	0.066 (2)	0.005 (2)	0.0023 (18)	−0.011 (2)
C12	0.068 (3)	0.133 (4)	0.075 (3)	0.000 (3)	0.011 (2)	−0.012 (3)
C13	0.066 (3)	0.169 (6)	0.100 (4)	0.033 (4)	0.000 (3)	−0.037 (4)
C14	0.075 (4)	0.124 (4)	0.113 (4)	0.048 (3)	−0.032 (3)	−0.040 (4)
C15	0.073 (3)	0.073 (3)	0.076 (3)	0.023 (2)	−0.027 (2)	−0.020 (2)
C16	0.112 (4)	0.067 (3)	0.098 (3)	0.013 (3)	−0.054 (3)	0.000 (3)
C17	0.119 (4)	0.077 (3)	0.073 (3)	−0.016 (3)	−0.046 (3)	0.020 (2)
C18	0.084 (3)	0.073 (2)	0.054 (2)	−0.010 (2)	−0.012 (2)	0.004 (2)
C19	0.066 (2)	0.0488 (19)	0.0512 (19)	−0.0036 (17)	−0.0095 (17)	0.0001 (17)
C20	0.056 (2)	0.057 (2)	0.0570 (19)	0.0089 (18)	−0.0102 (18)	−0.0111 (17)
C21	0.084 (3)	0.069 (3)	0.112 (3)	−0.012 (2)	−0.015 (3)	−0.023 (2)
C22	0.058 (2)	0.054 (2)	0.057 (2)	0.0021 (18)	0.0014 (17)	−0.0056 (17)
C23	0.051 (2)	0.055 (2)	0.0430 (17)	0.0017 (16)	0.0066 (15)	0.0018 (15)
C24	0.047 (2)	0.056 (2)	0.0495 (18)	0.0076 (17)	0.0057 (15)	−0.0022 (16)
C25	0.068 (3)	0.050 (2)	0.081 (3)	0.0092 (19)	−0.014 (2)	−0.0122 (19)
C26	0.069 (3)	0.061 (2)	0.086 (3)	0.016 (2)	−0.019 (2)	0.000 (2)
C27	0.047 (2)	0.056 (2)	0.054 (2)	0.0063 (17)	0.0044 (16)	0.0018 (17)
C28	0.050 (2)	0.059 (2)	0.061 (2)	0.0014 (19)	−0.0058 (19)	0.0064 (19)
C30	0.099 (4)	0.081 (3)	0.113 (4)	0.008 (3)	−0.055 (3)	−0.001 (3)
C31	0.109 (4)	0.072 (3)	0.152 (5)	−0.009 (3)	−0.057 (4)	−0.003 (3)
N1	0.0594 (19)	0.0587 (18)	0.0664 (19)	−0.0014 (15)	−0.0048 (15)	−0.0101 (15)
O1	0.076 (2)	0.109 (2)	0.099 (2)	−0.0064 (18)	0.0060 (17)	0.052 (2)
O2	0.0753 (18)	0.0601 (15)	0.0886 (18)	−0.0089 (15)	0.0040 (16)	0.0109 (14)
S1	0.0631 (6)	0.0538 (5)	0.0724 (6)	−0.0014 (5)	−0.0180 (5)	0.0079 (5)
S2	0.0851 (18)	0.0775 (18)	0.0916 (17)	0.0007 (15)	−0.0399 (16)	0.0029 (13)
C29'	0.094 (8)	0.099 (16)	0.123 (11)	0.021 (10)	−0.056 (7)	0.015 (10)
S2'	0.127 (5)	0.053 (3)	0.112 (4)	0.000 (3)	−0.073 (3)	0.010 (3)
C29	0.13 (2)	0.11 (2)	0.14 (2)	−0.036 (19)	−0.001 (17)	0.018 (17)
Cl1	0.1074 (10)	0.1174 (11)	0.1371 (12)	0.0501 (9)	−0.0110 (9)	−0.0470 (9)

### Geometric parameters (Å, °)

**Table:**

C1—C2	1.344 (7)	C17—H17	0.9300
C1—C6	1.349 (6)	C18—C19	1.360 (5)
C1—Cl1	1.748 (4)	C18—H18	0.9300
C2—C3	1.377 (6)	C19—C20	1.415 (5)
C2—H2	0.9300	C21—N1	1.450 (5)
C3—C4	1.375 (5)	C21—H21A	0.9600
C3—H3	0.9300	C21—H21B	0.9600
C4—C5	1.370 (5)	C21—H21C	0.9600
C4—C7	1.479 (5)	C22—N1	1.435 (4)
C5—C6	1.377 (6)	C22—C23	1.510 (5)
C5—H5	0.9300	C22—H22A	0.9700
C6—H6	0.9300	C22—H22B	0.9700
C7—O2	1.218 (4)	C23—C24	1.498 (4)
C7—C8	1.524 (5)	C23—H23	0.9800
C8—C23	1.535 (4)	C24—C25	1.354 (5)
C8—C9	1.577 (5)	C24—S1	1.723 (4)
C8—H8	0.9800	C25—C26	1.394 (5)
C9—N1	1.466 (4)	C25—H25	0.9300
C9—C19	1.517 (5)	C26—C27	1.332 (5)
C9—C10	1.573 (5)	C26—H26	0.9300
C10—O1	1.206 (4)	C27—C28	1.432 (5)
C10—C11	1.470 (5)	C27—S1	1.727 (3)
C11—C12	1.381 (5)	C28—C29'	1.396 (9)
C11—C20	1.396 (5)	C28—C29	1.402 (10)
C12—C13	1.387 (7)	C28—S2	1.682 (5)
C12—H12	0.9300	C28—S2'	1.703 (8)
C13—C14	1.360 (8)	C30—C31	1.318 (6)
C13—H13	0.9300	C30—C29	1.420 (10)
C14—C15	1.408 (7)	C30—S2	1.625 (6)
C14—H14	0.9300	C30—H30	0.9300
C15—C20	1.400 (5)	C31—C29'	1.416 (9)
C15—C16	1.403 (7)	C31—S2'	1.612 (9)
C16—C17	1.365 (7)	C31—H31	0.9300
C16—H16	0.9300	C29'—H29'	0.9300
C17—C18	1.419 (6)	C29—H29	0.9300
			
C2—C1—C6	120.5 (4)	C11—C20—C15	122.9 (4)
C2—C1—Cl1	120.4 (4)	C11—C20—C19	113.5 (3)
C6—C1—Cl1	119.2 (4)	C15—C20—C19	123.6 (4)
C1—C2—C3	120.9 (4)	N1—C21—H21A	109.5
C1—C2—H2	119.6	N1—C21—H21B	109.5
C3—C2—H2	119.6	H21A—C21—H21B	109.5
C4—C3—C2	120.2 (4)	N1—C21—H21C	109.5
C4—C3—H3	119.9	H21A—C21—H21C	109.5
C2—C3—H3	119.9	H21B—C21—H21C	109.5
C5—C4—C3	117.3 (4)	N1—C22—C23	102.3 (3)
C5—C4—C7	122.6 (3)	N1—C22—H22A	111.3
C3—C4—C7	120.1 (4)	C23—C22—H22A	111.3
C4—C5—C6	122.2 (4)	N1—C22—H22B	111.3
C4—C5—H5	118.9	C23—C22—H22B	111.3
C6—C5—H5	118.9	H22A—C22—H22B	109.2
C1—C6—C5	118.8 (4)	C24—C23—C22	114.4 (3)
C1—C6—H6	120.6	C24—C23—C8	114.5 (3)
C5—C6—H6	120.6	C22—C23—C8	101.9 (3)
O2—C7—C4	121.9 (3)	C24—C23—H23	108.6
O2—C7—C8	120.4 (3)	C22—C23—H23	108.6
C4—C7—C8	117.6 (3)	C8—C23—H23	108.6
C7—C8—C23	115.8 (3)	C25—C24—C23	128.9 (3)
C7—C8—C9	111.7 (3)	C25—C24—S1	109.0 (3)
C23—C8—C9	104.7 (2)	C23—C24—S1	122.1 (2)
C7—C8—H8	108.1	C24—C25—C26	114.0 (3)
C23—C8—H8	108.1	C24—C25—H25	123.0
C9—C8—H8	108.1	C26—C25—H25	123.0
N1—C9—C19	110.5 (3)	C27—C26—C25	114.6 (4)
N1—C9—C10	114.9 (3)	C27—C26—H26	122.7
C19—C9—C10	102.2 (3)	C25—C26—H26	122.7
N1—C9—C8	102.7 (3)	C26—C27—C28	128.7 (3)
C19—C9—C8	118.0 (3)	C26—C27—S1	109.5 (3)
C10—C9—C8	109.1 (3)	C28—C27—S1	121.8 (3)
O1—C10—C11	129.1 (4)	C29'—C28—C29	96.6 (8)
O1—C10—C9	122.9 (4)	C29'—C28—C27	132.2 (5)
C11—C10—C9	108.0 (3)	C29—C28—C27	130.4 (6)
C12—C11—C20	120.1 (4)	C29'—C28—S2	107.7 (6)
C12—C11—C10	132.3 (4)	C27—C28—S2	120.0 (3)
C20—C11—C10	107.5 (3)	C29—C28—S2'	106.7 (7)
C11—C12—C13	117.1 (5)	C27—C28—S2'	122.3 (3)
C11—C12—H12	121.5	S2—C28—S2'	117.5 (3)
C13—C12—H12	121.5	C31—C30—C29	104.2 (8)
C14—C13—C12	123.3 (5)	C31—C30—S2	117.8 (4)
C14—C13—H13	118.3	C31—C30—H30	121.1
C12—C13—H13	118.3	C29—C30—H30	133.1
C13—C14—C15	121.1 (4)	S2—C30—H30	121.1
C13—C14—H14	119.4	C30—C31—C29'	106.9 (6)
C15—C14—H14	119.4	C30—C31—S2'	120.4 (5)
C20—C15—C16	116.4 (4)	C30—C31—H31	126.5
C20—C15—C14	115.4 (4)	C29'—C31—H31	126.5
C16—C15—C14	128.2 (5)	S2'—C31—H31	113.0
C17—C16—C15	120.6 (4)	C22—N1—C21	115.5 (3)
C17—C16—H16	119.7	C22—N1—C9	109.4 (3)
C15—C16—H16	119.7	C21—N1—C9	116.2 (3)
C16—C17—C18	121.9 (5)	C24—S1—C27	92.86 (17)
C16—C17—H17	119.0	C30—S2—C28	92.3 (3)
C18—C17—H17	119.0	C28—C29'—C31	114.8 (8)
C19—C18—C17	119.3 (4)	C28—C29'—H29'	122.6
C19—C18—H18	120.3	C31—C29'—H29'	122.6
C17—C18—H18	120.3	C31—S2'—C28	91.2 (5)
C18—C19—C20	118.2 (3)	C28—C29—C30	115.5 (10)
C18—C19—C9	133.1 (4)	C28—C29—H29	122.3
C20—C19—C9	108.7 (3)	C30—C29—H29	122.3
			
C6—C1—C2—C3	1.7 (8)	N1—C22—C23—C24	−166.4 (3)
Cl1—C1—C2—C3	−177.1 (4)	N1—C22—C23—C8	−42.3 (3)
C1—C2—C3—C4	−2.0 (8)	C7—C8—C23—C24	−83.9 (4)
C2—C3—C4—C5	0.1 (6)	C9—C8—C23—C24	152.7 (3)
C2—C3—C4—C7	−178.5 (4)	C7—C8—C23—C22	152.0 (3)
C3—C4—C5—C6	2.1 (7)	C9—C8—C23—C22	28.6 (3)
C7—C4—C5—C6	−179.4 (4)	C22—C23—C24—C25	1.3 (5)
C2—C1—C6—C5	0.5 (8)	C8—C23—C24—C25	−115.8 (4)
Cl1—C1—C6—C5	179.3 (4)	C22—C23—C24—S1	−179.3 (2)
C4—C5—C6—C1	−2.4 (7)	C8—C23—C24—S1	63.6 (4)
C5—C4—C7—O2	151.1 (4)	C23—C24—C25—C26	−179.9 (3)
C3—C4—C7—O2	−30.3 (5)	S1—C24—C25—C26	0.7 (4)
C5—C4—C7—C8	−32.4 (5)	C24—C25—C26—C27	−0.2 (5)
C3—C4—C7—C8	146.1 (4)	C25—C26—C27—C28	−179.7 (3)
O2—C7—C8—C23	−12.0 (5)	C25—C26—C27—S1	−0.4 (5)
C4—C7—C8—C23	171.5 (3)	C26—C27—C28—C29'	176.8 (17)
O2—C7—C8—C9	107.7 (4)	S1—C27—C28—C29'	−2.5 (17)
C4—C7—C8—C9	−68.8 (4)	C26—C27—C28—C29	10 (2)
C7—C8—C9—N1	−131.0 (3)	S1—C27—C28—C29	−170 (2)
C23—C8—C9—N1	−5.0 (3)	C26—C27—C28—S2	−5.6 (6)
C7—C8—C9—C19	−9.3 (4)	S1—C27—C28—S2	175.2 (3)
C23—C8—C9—C19	116.7 (3)	C26—C27—C28—S2'	180.0 (8)
C7—C8—C9—C10	106.6 (3)	S1—C27—C28—S2'	0.8 (8)
C23—C8—C9—C10	−127.3 (3)	C29—C30—C31—C29'	8(2)
N1—C9—C10—O1	−62.6 (5)	S2—C30—C31—C29'	−4.8 (15)
C19—C9—C10—O1	177.7 (4)	C29—C30—C31—S2'	10.7 (18)
C8—C9—C10—O1	52.1 (5)	S2—C30—C31—S2'	−1.8 (11)
N1—C9—C10—C11	117.3 (3)	C23—C22—N1—C21	175.1 (3)
C19—C9—C10—C11	−2.4 (4)	C23—C22—N1—C9	41.8 (3)
C8—C9—C10—C11	−128.1 (3)	C19—C9—N1—C22	−149.3 (3)
O1—C10—C11—C12	−0.1 (8)	C10—C9—N1—C22	95.7 (3)
C9—C10—C11—C12	−180.0 (4)	C8—C9—N1—C22	−22.7 (3)
O1—C10—C11—C20	−177.7 (4)	C19—C9—N1—C21	77.8 (4)
C9—C10—C11—C20	2.5 (4)	C10—C9—N1—C21	−37.2 (4)
C20—C11—C12—C13	−0.8 (6)	C8—C9—N1—C21	−155.6 (3)
C10—C11—C12—C13	−178.2 (4)	C25—C24—S1—C27	−0.7 (3)
C11—C12—C13—C14	−0.2 (8)	C23—C24—S1—C27	179.8 (3)
C12—C13—C14—C15	0.8 (8)	C26—C27—S1—C24	0.6 (3)
C13—C14—C15—C20	−0.4 (6)	C28—C27—S1—C24	180.0 (3)
C13—C14—C15—C16	−179.6 (5)	C31—C30—S2—C28	6.1 (6)
C20—C15—C16—C17	−0.2 (6)	C29—C30—S2—C28	−37 (4)
C14—C15—C16—C17	179.0 (4)	C29'—C28—S2—C30	−5.1 (13)
C15—C16—C17—C18	0.3 (7)	C29—C28—S2—C30	42 (4)
C16—C17—C18—C19	0.2 (6)	C27—C28—S2—C30	176.7 (3)
C17—C18—C19—C20	−0.8 (5)	S2'—C28—S2—C30	−8.6 (8)
C17—C18—C19—C9	−178.5 (4)	C29—C28—C29'—C31	−8(2)
N1—C9—C19—C18	56.5 (5)	C27—C28—C29'—C31	−178.6 (9)
C10—C9—C19—C18	179.3 (4)	S2—C28—C29'—C31	4(2)
C8—C9—C19—C18	−61.1 (5)	S2'—C28—C29'—C31	166 (12)
N1—C9—C19—C20	−121.3 (3)	C30—C31—C29'—C28	0(2)
C10—C9—C19—C20	1.5 (3)	S2'—C31—C29'—C28	−169 (9)
C8—C9—C19—C20	121.1 (3)	C30—C31—S2'—C28	−3.6 (11)
C12—C11—C20—C15	1.3 (6)	C29'—C31—S2'—C28	8(7)
C10—C11—C20—C15	179.2 (3)	C29'—C28—S2'—C31	−11 (9)
C12—C11—C20—C19	−179.5 (4)	C29—C28—S2'—C31	−5.2 (19)
C10—C11—C20—C19	−1.6 (4)	C27—C28—S2'—C31	−177.6 (4)
C16—C15—C20—C11	178.7 (4)	S2—C28—S2'—C31	7.8 (10)
C14—C15—C20—C11	−0.6 (5)	C29'—C28—C29—C30	14 (3)
C16—C15—C20—C19	−0.5 (5)	C27—C28—C29—C30	−175.8 (12)
C14—C15—C20—C19	−179.8 (4)	S2—C28—C29—C30	−121 (6)
C18—C19—C20—C11	−178.2 (3)	S2'—C28—C29—C30	13 (3)
C9—C19—C20—C11	0.0 (4)	C31—C30—C29—C28	−15 (3)
C18—C19—C20—C15	1.0 (5)	S2—C30—C29—C28	127 (6)
C9—C19—C20—C15	179.2 (3)		
